# Molecular genetics of Iranian patients with acid sphingomyelinase deficiency and Niemann-Pick disease type C: A comparative case series

**DOI:** 10.1016/j.ymgmr.2026.101380

**Published:** 2026-11-07

**Authors:** Sheyda Khalilian, Mohadeseh Fathi, Shadab Salehpour, Davoud Amirkashani, Saeed Talebi, Soudeh Ghafouri-Fard, Mohammad Miryounesi

**Affiliations:** a Student Research Committee, School of Medicine, Shahid Beheshti University of Medical Sciences, Tehran, Iran; b Department of Medical Genetics, Shahid Beheshti University of Medical Sciences, Tehran, Iran; c Department of Pediatrics, Clinical Research Development Unit, Loghman Hakim Hospital, Shahid Beheshti University of Medical Sciences, Tehran, Iran; d Pediatric Endocrinology Department, Ali Asghar Children Hospital, Iran University of Medical Sciences, Tehran, Iran; e Department of Medical Genetics, School of Medicine, Iran University of Medical Sciences (IUMS), Tehran, Iran; f Genomic Research Center, Shahid Beheshti University of Medical Sciences, Tehran, Iran

**Keywords:** Acid sphingomyelinase deficiency, Niemann-Pick disease type C, SMPD1, NPC1, NPC2, Iran

## Abstract

**Background:**

Acid sphingomyelinase deficiency (ASMD), historically termed Niemann-Pick disease types A and B, and Niemann-Pick disease type C (NPC) represent distinct lysosomal storage disorders with different molecular etiologies. ASMD results from SMPD1 mutations causing sphingomyelinase deficiency, whereas NPC arises from NPC1 or NPC2 mutations disrupting intracellular cholesterol trafficking. Despite their historical grouping under “Niemann-Pick disease,” these conditions are genetically, biochemically, and clinically distinct.

**Methods:**

This study investigated 11 unrelated Iranian individuals referred for genetic testing with suspected NPD (Niemann-Pick disease). Whole exome sequencing (WES) was performed, and variants were classified according to ACMG guidelines.

**Results:**

Pathogenic or likely pathogenic variants were identified in 10 of 11 cases. SMPD1 variants (ASMD) were detected in six cases, with truncating variants associated with severe neuronopathic presentations (type A) and missense variants associated with predominantly visceral involvement (type B). NPC1 variants were identified in three cases, and NPC2 variants in two cases. Homozygosity was observed in 10 of 11 cases, consistent with autosomal recessive inheritance and the high rate of consanguinity (63.6%) in this cohort.

**Conclusion:**

This study provides a comparative genetic analysis of ASMD and NPC in the Iranian population, highlighting the variant spectrum and genotype-phenotype correlations within each distinct disorder. The findings underscore the importance of molecular diagnosis for accurate classification and genetic counseling, particularly in populations with high consanguinity.

## 1 Introduction

The term “Niemann-Pick disease” (NPD) historically encompasses a group of rare inherited lysosomal storage disorders characterized by the accumulation of lipids within lysosomes. However, advances in molecular genetics have revealed that these conditions are fundamentally distinct at the biochemical and genetic levels. Acid sphingomyelinase deficiency (ASMD), historically referred to as Niemann-Pick disease types A and B, results from mutations in the SMPD1 gene, leading to deficiency of acid sphingomyelinase and subsequent accumulation of sphingomyelin [1]. In contrast, Niemann-Pick disease type C (NPC) arises from mutations in either NPC1 or NPC2, disrupting intracellular cholesterol trafficking and leading to progressive neurodegeneration [2], [3], [4]. ASMD presents as a phenotypic spectrum. Type A (acute neuronopathic form) is characterized by early onset, rapid neurodegenerative progression, significant hepatosplenomegaly, and typically results in death by age three years [5]. Type B (chronic visceral form) manifests with prominent hepatosplenomegaly with variable or absent neurological involvement, and affected individuals often survive into adulthood, especially with enzyme replacement therapy. The classification of types A and B as distinct entities is increasingly replaced by a spectrum model of ASMD severity [6]. NPC, the most common form historically grouped under NPD, exhibits variable onset typically during childhood or adolescence and is marked by neurological deterioration with distinctive features including vertical supranuclear gaze palsy (VSGP), hypotonia, motor retardation, gelastic cataplexy, dystonia, fine and gross motor impairment, speech and swallowing difficulties, and progressive cognitive decline [7]. Systemic manifestations including hepatosplenomegaly may also occur, though often less prominent than in ASMD [2], [8].

The distinction between ASMD and NPC is clinically critical, as their management strategies differ substantially [9], [10]. Enzyme replacement therapy (Olipudase Alfa) is now available for ASMD, whereas NPC management remains largely supportive, though Miglustat may slow neurological progression [11], [12]. Beyond Miglustat, recent regulatory approvals for *N*-acetyl-L-leucine (in the US and UK) and Arimoclomol (in the US) have expanded therapeutic options for NPC, with several other agents currently in clinical trials. Accurate molecular diagnosis is therefore essential for appropriate patient management and genetic counseling.

Consanguinity is highly prevalent in Iran, with rates of 30–40% in some regions, increasing the likelihood of homozygous recessive conditions [13]. This demographic context underscores the importance of characterizing the molecular spectrum of autosomal recessive disorders in the Iranian population.

This study presents a comparative molecular genetic analysis of Iranian patients with ASMD and NPC, describing the variant spectrum and genotype-phenotype correlations within each distinct disorder.

## 2 Materials and methods

### 2.1 Case presentation

The research cohort comprised 11 unrelated Iranian patients with suspected Niemann-Pick disease (ASMD or NPC), ascertained via consecutive referrals to the Comprehensive Genomic Center between 2018 and 2024. The study was approved by the Ethics Committee of Shahid Beheshti University of Medical Sciences. All procedures followed the Declaration of Helsinki guidelines. Informed consent forms were signed by legal representatives of all patients. The application of the study's inclusion and exclusion criteria was as follows:

Inclusion Criteria

1. Diagnostic suspicion of ASMD or NPC based on clinical, biochemical, and/or histopathological findings.
2. Referral for genetic testing due to suspected NPD.
3. Availability of WES data with sufficient quality for variant analysis.
4. Clinical and/or laboratory records supporting the phenotypic presentation (e.g., hepatosplenomegaly, neurological involvement, lysosomal enzyme deficiency, or abnormal lipid storage).
5. Informed consent for genetic testing and research use of data.

Exclusion Criteria

1. Confirmed alternative genetic diagnoses explaining the phenotype (e.g., other lysosomal storage disorders, Gaucher disease).
2. Incomplete clinical or laboratory data preventing accurate classification
3. Poor-quality WES data (low coverage, high missingness) that precludes reliable variant calling.
4. Insufficient consent for research use of genetic data.

### 2.2 Molecular analyses

Genomic DNA was isolated from peripheral blood samples of probands using the standard salting-out protocol. DNA purity and concentration were assessed using a NanoDrop 1000 spectrophotometer (Thermo Fisher Scientific, USA). WES was performed on an Illumina HiSeq4000 platform, generating 101-bp paired-end reads with a minimum coverage of 100×. Exonic regions and flanking splice sites were captured using the SureSelectXT2 V6 enrichment kit. Raw sequencing data underwent quality control, including the removal of low-quality reads, followed by alignment to the human reference genome (GRCh37/hg19) using the Burrows-Wheeler Aligner (BWA). Duplicate reads were flagged and removed using SAMtools. Base quality recalibration and variant calling (SNPs/indels) were conducted using the Genome Analysis Toolkit (GATK v4). For variant prioritization, an initial manual filtering step was applied. Known disease-associated variants in ClinVar were cross-referenced with the patient's phenotype, and inheritance patterns were evaluated. Finally, variants were functionally annotated and classified using ANNOVAR. Segregation analyses were conducted in the families that demanded prenatal diagnosis for later conceptions.

## 3 Results

### 3.1 Clinical characteristics

The study included 11 patients with NPD (Table 1), ranging in age from 3 months to 23 years, with a predominance of males (6/11, 54.5%). Consanguinity was reported in 7 cases (63.6%), consistent with autosomal recessive inheritance and the high rate of consanguineous unions in Iran. Hepatosplenomegaly was the most common feature, present in 7/9 patients (77%) with available data (two cases lacked documentation). Developmental delay was observed in 4/7 patients (57.1%) where assessable (Cases 1, 8, 9, 10), while speech delay was noted in Case 1. Seizures were reported in 2 patients (Cases 9 and 10). Cherry-red spot, a classic ocular finding historically associated with ASMD type A, was documented in only Case 1. Short stature was present in Cases 5.

**Table 1:** Summary of clinical data of study patients.

Case Number	Age/ Sex	ASMD/NPC	Consanguinity	Motor delay	Cognitive delay	Seizure	Speech delay	VSGP	Hepatosplenomegaly	Cherry red spot
1	15 months/ Male	ASMD	Yes	+	+	_	+	N/A	+	+
2	11 years/ Male	ASMD	No	N/A	N/A	_	N/A	N/A	+	_
3	4 years/ Male	ASMD	Yes	N/A	N/A	_	N/A	N/A	+	_
4	6 years/ Female	ASMD	Yes	N/A	N/A	_	N/A	N/A	+	_
5	2 years/ Female	ASMD	Yes	N/A	N/A	_	N/A	N/A	+	_
6	23 years/ Male	ASMD	No	−	−	_	−	−	+	_
7	3.5 years/ Female	NPC1	Yes	N/A	N/A	_	N/A	N/A	+	_
8	3 years/ Female	NPC1	Yes	+	−	_	N/A	N/A	N/A	_
9	5 years/ Male	NPC1	No	+	−	+	N/A	N/A	N/A	_
10	3.5 years/ Female	NPC2	Yes	+	−	+	_	N/A	_	_
11	3 months/ Male	NPC2	Yes	N/A	N/A	_	_	N/A	_	_

N/A: Not available/not assessed; +: present; −: absent.

Notably, Case 11 (3-month-old male with *NPC2* variant) exhibited none of the typical features (no hepatosplenomegaly, cherry red spot, or growth abnormalities), highlighting phenotypic variability and potential for later-onset manifestations. Developmental and speech assessments were not available (N/A) for younger patients, likely due to age-related limitations. This individual was referred for genetic testing due to a positive family history of NPC2 in an older sibling, and was therefore pre-symptomatic at the time of evaluation. This highlights the phenotypic variability and the importance of early molecular screening within affected families.

Fig. 1 shows the frequency of major clinical manifestations in the cohort as a bar chart.

**Fig. 1:**
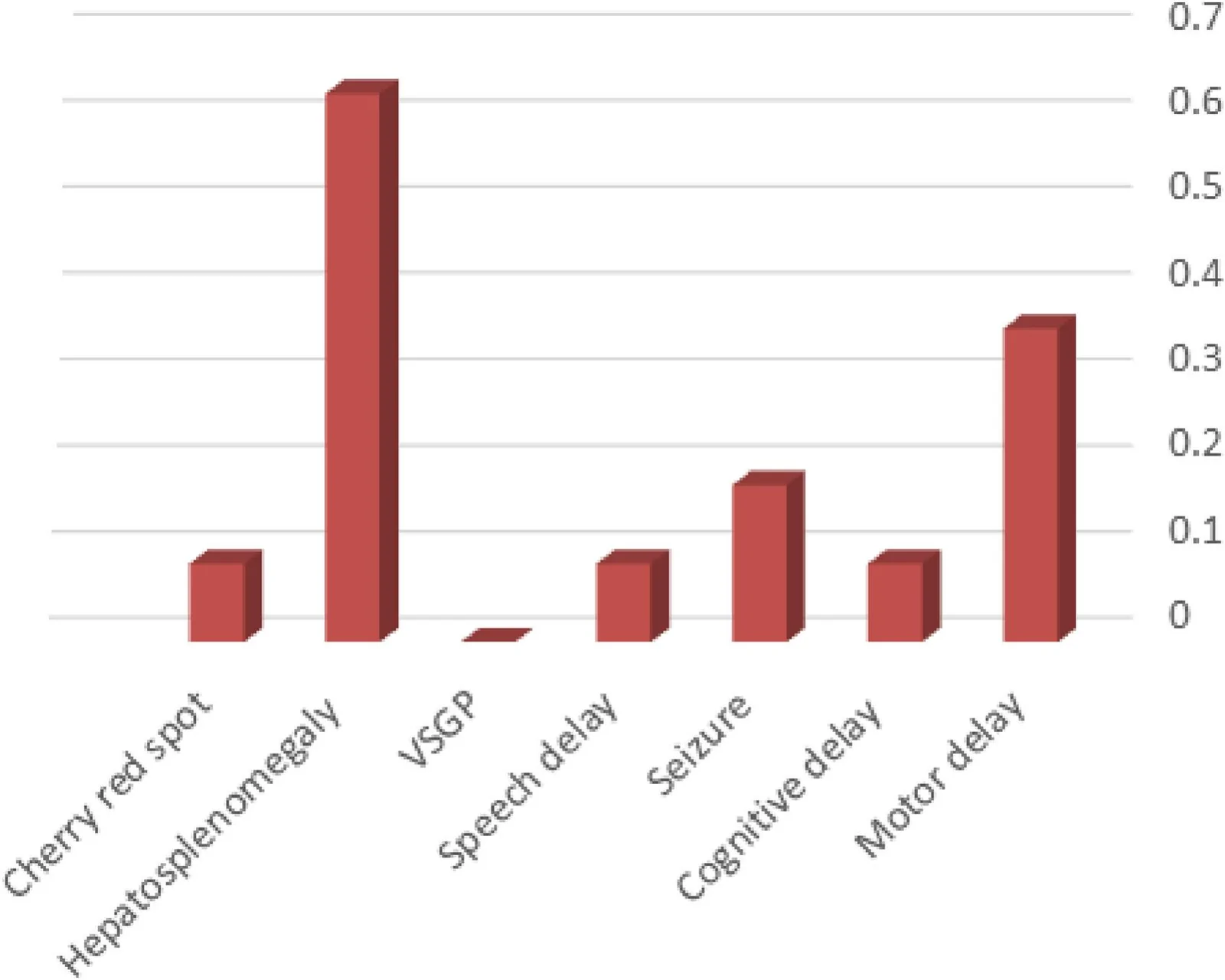
Bar chart showing the frequency of major clinical manifestations in the cohort.

### 3.2 Molecular findings

Pathogenic and likely pathogenic variants were identified in 10 of 11 patients, involving SMPD1 (ASMD), NPC1 (NPC type C1), and NPC2 (NPC type C2) (Table 2). Most variants were homozygous (10/11 cases), consistent with autosomal recessive inheritance. Case 9 was compound heterozygous for two variants of uncertain significance (VUS).

**Table 2:** Summary of molecular data of study patients.

Case number	Gene	Transcript	Variant	Associated disease	OMIM	Inheritance	Zygosity	ACMG classification	ClinVar	ACMG Evidence Codes	Ref
1	*SMPD1*	NM_000543.5	c.528G > A p.W176X	NPD, type A NPD, type B	257200 607616	AR AR	Hom	Pathogenic	Pathogenic/Likely pathogenic	PM3, PVS1, PM2, PP5	–
2	*SMPD1*	NM_000543.5	c.1244C > T p.A415V	NPD, type A NPD, type B	257200 607616	AR AR	Hom	Likely Pathogenic	Pathogenic/ VUS	PM1, PP2, PM2, PP3	–
3	*SMPD1*	NM_000543	c.416 T > C p.L139P	NPD, type A NPD type B	257200 607616	AR AR	Hom	Likely Pathogenic	Pathogenic/Likely pathogenic	PM3, PM1, PP2, PM2, PP3, PP5	–
4	*SMPD1*	NM_000543	c.1556 A > G p.Y519C	NPD, type A NPD, type B	257200 607616	AR AR	Hom	Pathogenic	Conflict (Pathogenic/Likely pathogenic/VUS)	PM1, PP2, PM2, PP3, PP5,	–
5	*SMPD1*	NM_000543	c.416 T > C p.L139P	NPD type A NPD, type B	257200 607616	AR AR	Hom	Likely Pathogenic	Pathogenic/Likely pathogenic	PM3, PM1, PP2, PM2, PP3, PP5	–
6	*SMPD1*	NM_000543	c.416 T > C p.L139P	NPD, type A NPD, type B	257200 607616	AR AR	Hom	Likely Pathogenic	Pathogenic/Likely pathogenic	PM3, PM1, PP2, PM2, PP3, PP5	–
7	*NPC1*	NM_000271.5	c.456dup p.A153fs	NPD, type C1 NPD, type D	257220 257220	AR AR	Hom	Likely Pathogenic	N/R	PVS1, PM2	–
8	*NPC1*	NM_000271.5	c.3051_3066del p.A1018Lfs*17	NPD, type C1 NPD, type D	257220 257220	AR AR	Hom	Likely Pathogenic	N/R	PVS1, PM2	–
9	*NPC1*	NM_000271.5	c.541G > A p.A181T	NPD, type C1 NPD, type D	257220 257220	AR AR	Het	VUS	VUS	PM2, PM1, PP2	[14]
			c.83 A > G p.Y28C				Het	VUS	VUS	PM2, PP3, PP2	–
10	*NPC2*	NM_006432.5	c.279dup p.K94Ifs*5	NPD, type C2	607625	AR	Hom	Pathogenic	Pathogenic/Likely pathogenic	PVS1, PM2, PP5	–
	*FMN2*	NM_020066.5	c.2840del p.P947Lfs*328	Intellectual developmental disorder, autosomal recessive 47	616193	AR	Hom	VUS	Likely pathogenic	PVS1, PP5	–
11	*NPC2*	NM_006432	c.157C > T p.Q53X	NPD, type C2	607625	AR	Hom	Pathogenic	Pathogenic	PM3, PVS1, PM2, PP5	–

#### 3.2.1 Acid Sphingomyelinase deficiency (SMPD1 variants)

*SMPD1* variants were detected in six cases (Cases 1–6). Case 1 was homozygous for the nonsense variant c.528G > A (p.W176X), classified as pathogenic (ACMG). This truncating variant was associated with severe infantile-onset disease (type A phenotype), with developmental delay, hepatosplenomegaly, and cherry-red spot by 15 months.

Case 2 was homozygous for c.1244C > T (p.A415V) (likely pathogenic), presenting with hepatosplenomegaly without neurological symptoms at age 11 years, consistent with type B phenotype.

Cases 3, 5, and 6 were homozygous for the missense variant c.416 T > C (p.L139P), classified as likely pathogenic. All three cases presented with hepatosplenomegaly without documented neurological involvement, consistent with ASMD type B. The recurrence of this variant in three unrelated families suggests a possible founder effect in the Iranian population, though its clinical spectrum requires further validation.

Case 4 was homozygous for c.1556 A > G (p.Y519C) (pathogenic, CADD 23). This variant has conflicting interpretations in ClinVar (pathogenic/likely pathogenic/VUS), highlighting the need for functional validation.

#### 3.2.2 Niemann-Pick disease type C (NPC1 variants)

*NPC1* variants were detected in Cases 7–9. Case 7 was homozygous for the frameshift variant c.456dup (p.A153fs), classified as likely pathogenic. Case 8 was homozygous for the frameshift variant c.3051_3066del (p.A1018Lfs*17), classified as likely pathogenic. Case 9 was compound heterozygous for two missense VUS: c.541G > A (p.A181T) and c.83 A > G (p.Y28C). This patient presented with developmental delay and seizures, which are consistent with NPC, though the variants require functional validation to confirm pathogenicity.

#### 3.2.3 Niemann-Pick disease type C (NPC2 variants)

*NPC2* variants were detected in Cases 10 and 11. Case 10 was homozygous for the frameshift variant c.279dup (p.K94Ifs*5), classified as pathogenic. This patient also carried a homozygous FMN2 variant (c.2840del, p.P947Lfs*328) classified as VUS, which may contribute to the severe developmental delay observed beyond typical NPC2 expectations (OMIM 616193). Case 11 (3-month-old male) was homozygous for the nonsense variant c.157C > T (p.Q53X), classified as pathogenic. Notably, this patient had no hepatosplenomegaly or cherry-red spot at presentation, suggesting either later-onset manifestations or a milder phenotype.

### 3.3 Genotype-phenotype correlations

#### 3.3.1 ASMD (*SMPD1*)

In patients with *SMPD1* variants, a clear variant-phenotype stratification was observed:

• Truncating variants (p.W176X, Case 1) were associated with severe infantile-onset ASMD (type A phenotype) characterized by rapid neurodegeneration, hepatosplenomegaly, and cherry-red spot.
• Missense variants (p.L139P, p.A415V, p.Y519C; Cases 2–6) were predominantly associated with visceral involvement (hepatosplenomegaly) without documented neurological symptoms, consistent with ASMD type B. These patients had later onset and, in Case 6, survival into adulthood (23 years).

The p.L139P variant's recurrence in three unrelated cases suggests a possible founder effect in the Iranian population, though variable expressivity was observed (Cases 3, 5, and 6 lacked neurological signs).

#### 3.3.2 NPC (*NPC1* and *NPC2*)

• Frameshift variants in *NPC1* (Cases 7–8) were classified as likely pathogenic and are expected to cause severe NPC type C1 with neurovisceral involvement, though detailed clinical data were limited.
• Case 9 with compound heterozygous VUS in *NPC1* presented with developmental delay and seizures, consistent with NPC, but the variants require functional validation.
• *NPC2* variants (Cases 10–11) resulted in truncating mutations with variable presentation. For NPC2, cherry-red spots are exceedingly rare, and hepatosplenomegaly is less prominent, with pulmonary involvement and neurological manifestations being more characteristic. Case 11 (p.Q53X) had no hepatosplenomegaly or cherry-red spot at 3 months, that is consistent with his early age and positive family history, and suggesting that systemic manifestations may appear later.
• Comorbid *FMN2* variant (Case 10) may explain the severe developmental delay beyond NPC2 expectations.

## 4 Discussion

This study presents a comparative genetic analysis of ASMD and NPC in 11 Iranian patients, identifying pathogenic or likely pathogenic variants in 10 cases. While these disorders share historical nomenclature, they represent distinct molecular entities with different pathogenic mechanisms—lysosomal sphingomyelinase deficiency in ASMD versus defective cholesterol trafficking in NPC. The findings highlight the genetic heterogeneity within each disorder and the importance of molecular diagnosis for accurate classification, prognostic counseling, and therapeutic decision-making. A previous study among 19 Iranian cases of NPD, has presented 9 formerly reported mutations and 10 novel mutations [15]. Notably, none of these variants were detected in our cohort, highlighting the heterogeneity of NPD among Iranian patients.

### 4.1 ASMD variant spectrum and phenotype correlations

In our cohort, *SMPD1* variants demonstrated clear genotype-phenotype correlations. The truncating variant p.W176X (Case 1) was associated with severe infantile-onset ASMD (type A phenotype), consistent with the established concept that null alleles result in complete enzyme deficiency and severe neurological involvement [16]. In contrast, missense variants (p.L139P, p.A415V, p.Y519C) were associated with predominantly visceral involvement (type B phenotype), consistent with residual enzyme activity sufficient to prevent or delay neurological manifestations [17].

The recurrence of p.L139P in three unrelated Iranian families is noteworthy. Notably, in our cohort, this variant was associated with a predominantly visceral phenotype without documented neurological involvement, contrasting with the classic severe neuronopathic forms often associated with null alleles. To date, no definitive published case series has described the precise phenotypic spectrum of this specific variant in detail, making our observation valuable for genotype-phenotype correlation. The variability in our cases may reflect differences in genetic background, modifier genes, or environmental factors, and warrants further investigation in larger population-based studies.

The conflicting ClinVar interpretations for p.Y519C (Case 4) highlight the ongoing challenges in variant classification for rare diseases. Functional studies, including enzyme activity assays in patient fibroblasts, would strengthen pathogenicity evidence for this and other variants with uncertain classification.

### 4.2 Homozygosity in non-consanguineous families

While most homozygous variants in this cohort occurred in the context of consanguineous unions (7/10 homozygous cases), Case 2 was homozygous for the *SMPD1* c.1244C > T variant despite no reported consanguinity. Homozygous variants in non-consanguineous families can occur due to localized carrier frequencies, particularly when a variant is relatively common in a specific population. This observation underscores the importance of segregation analysis for accurate genetic interpretation.

### 4.3 NPC variant spectrum

The *NPC1* frameshift variants identified in Cases 7–8 (p.A153fs, p.A1018Lfs*17) are predicted to result in complete loss of function, consistent with severe NPC type C1. However, the limited clinical data for these cases precludes detailed phenotype correlation.

Case 9, with compound heterozygous VUS in *NPC1*, presents a diagnostic challenge. While the clinical presentation (developmental delay and seizures) is consistent with NPC, functional validation of these variants through plasma oxysterols, bile acids, and Filipin staining of fibroblasts is required to confirm pathogenicity. This case illustrates the importance of integrating molecular findings with biochemical testing.

The *NPC2* variants identified in Cases 10–11 (p.K94Ifs*5, p.Q53X) are both truncating mutations predicted to result in complete loss of NPC2 protein. The variable presentation—with Case 11 showing no manifestations at 3 months—highlights the phenotypic variability of NPC2 deficiency and the potential for later-onset systemic involvement. This case emphasizes the clinical value of early WES screening, as identification of a pathogenic variant prior to symptom onset enables proactive, long-term clinical monitoring and facilitates early intervention when symptoms emerge.

### 4.4 Comorbid conditions and diagnostic complexity

Case 10, who carried a homozygous FMN2 variant in addition to the NPC2 variant, illustrates the complexity of genetic diagnosis in patients with atypical or severe phenotypes. FMN2 mutations are associated with autosomal recessive intellectual developmental disorder 47 (OMIM 616193). The severe developmental delay observed in this case may be attributable to the combined effects of both genetic variants, rather than NPC2 deficiency alone. This finding underscores the importance of comprehensive genetic analysis in patients with complex phenotypes, as comorbid conditions may influence disease presentation and management.

### 4.5 Clinical implications and therapeutic considerations

The distinction between ASMD and NPC has significant clinical implications. Enzyme replacement therapy with Olipudase Alfa is now available for ASMD, offering disease-modifying treatment for affected individuals [11]. In contrast, NPC management remains largely supportive, though Miglustat may slow neurological progression in some patients [12]. Accurate molecular diagnosis is therefore essential for appropriate treatment selection and patient counseling.

For families with affected individuals, particularly in populations with high consanguinity such as Iran, molecular diagnosis enables accurate genetic counseling, carrier screening, and prenatal diagnosis for subsequent pregnancies. Early diagnosis through WES also enables proactive monitoring, particularly for infants like Case 11 who carry severe pathogenic variants but have not yet developed clinical manifestations.

### 4.6 Limitations and future directions

This study has several limitations. First, the sample size is small, limiting the generalizability of genotype-phenotype correlations. Second, clinical data were incomplete for many patients, particularly regarding neurological examinations, ophthalmologic assessments, pulmonary function, and longitudinal follow-up. Third, biochemical testing (e.g., plasma oxysterols, chitotriosidase activity, and fibroblast Filipin staining) was not systematically performed for all patients prior to or alongside WES in this retrospective cohort. These tests are standard diagnostic tools and would have provided critical functional correlation, especially for variants of uncertain significance and the pre-symptomatic Case 11. Future studies should incorporate biochemical testing as a first-line diagnostic step combined with molecular analysis. Fourth, the potential founder effect for p.L139P requires validation in larger population studies. Fifth, given that WES was performed on probands rather than trios in most families, we could not formally exclude de novo variants or uniparental isodisomy. Finally, the absence of systematic biochemical testing (e.g., plasma oxysterols, Filipin staining) for all patients limits the ability to correlate molecular findings with biochemical parameters. Despite these limitations, this study provides valuable insights into the molecular spectrum of ASMD and NPC in the Iranian population and highlights the importance of molecular diagnosis in regions with high consanguinity.

Future studies should focus on prospective clinical characterization of affected individuals, including standardized neurological, ophthalmologic, and pulmonary assessments. Functional validation of VUS through fibroblast studies and enzyme activity assays will strengthen genotype-phenotype correlations. Population-based screening for recurrent variants (e.g., p.L139P) may inform carrier screening programs in Iran. Additionally, longitudinal follow-up of patients will help clarify disease progression and genotype-outcome relationships.

## 5 Conclusions

This study provides a comparative molecular genetic analysis of ASMD and NPC in Iranian patients, highlighting the distinct genetic etiologies and variant spectra of these historically grouped disorders. Clear genotype-phenotype correlations were observed in ASMD, with truncating variants associated with severe neuronopathic disease and missense variants with predominantly visceral involvement. The identification of recurrent and novel variants underscores the genetic heterogeneity of these conditions in the Iranian population. Molecular diagnosis is essential for accurate classification, prognostic counseling, and therapeutic decision-making, particularly in populations with high consanguinity.

## Authors' contributions

S.K. and M.F. evaluated patients' genetic reports. S.GF wrote the manuscript. M.M, S·S, D.A, S.T. assessed the patients. S.GF, and MM supervised the study. All the authors read and approved the submitted version.

## CRediT authorship contribution statement

**Sheyda Khalilian:** Investigation. **Mohadeseh Fathi:** Investigation. **Shadab Salehpour:** Resources. **Davoud Amirkashani:** Resources. **Saeed Talebi:** Resources. **Soudeh Ghafouri-Fard:** Writing – review & editing, Writing – original draft, Supervision. **Mohammad Miryounesi:** Supervision.

## Consent to participate

Informed consent forms were signed by legal representatives of patients.

## Consent to publish

Informed consent forms were signed by legal representatives of patients.

## Compliance with ethical standards

Informed consent forms were signed by legal representatives of patients. All methods were carried out in accordance with relevant guidelines and regulations. All experimental protocols were approved by ethical committee of Shahid Beheshti University of Medical Sciences.

## Funding

Not applicable.

## Declaration of competing interest

Authors declare no conflict of interests.

## Data Availability

The datasets generated and/or analysed during the current study are available in the Clinvar repository (https://www.ncbi.nlm.nih.gov/clinvar/?term=SMPD1+%5Bgene%5D) under accession numbers: VCV000552566.11, SCV004805335.2, VCV000203424.11, VCV000437453.10, VCV000497961.13, VCV001696117.3 and VCV000915429.3.
